# Effects of a Very Low-Carbohydrate High-Fat Diet and High-Intensity Interval Training on Visceral Fat Deposition and Cardiorespiratory Fitness in Overfat Individuals: A Randomized Controlled Clinical Trial

**DOI:** 10.3389/fnut.2021.785694

**Published:** 2021-12-21

**Authors:** Lukas Cipryan, Tomas Dostal, Martina Litschmannova, Peter Hofmann, Philip B. Maffetone, Paul B. Laursen

**Affiliations:** ^1^Department of Human Movement Studies & Human Motion Diagnostic Centre, The University of Ostrava, Ostrava, Czechia; ^2^Department of Applied Mathematics, VSB – Technical University of Ostrava, Ostrava, Czechia; ^3^Institute of Human Movement Science, Sport & Health, Exercise Physiology, Training & Training Therapy Research Group, University of Graz, Graz, Austria; ^4^Independent Researcher, Ormond Beach, FL, United States; ^5^Sports Performance Research Institute New Zealand (SPRINZ), Auckland University of Technology, Auckland, New Zealand

**Keywords:** carbohydrates, overfat, exercise, body composition, fitness level, health

## Abstract

**Purpose:** This randomized controlled parallel-group study examined the effects of a very low-carbohydrate high-fat (VLCHF) diet and high-intensity interval training (HIIT) program over 12 weeks on visceral adipose tissue (VAT) and cardiorespiratory fitness (CRF) level in overfat individuals.

**Methods:** Ninety-one participants were randomly allocated to the HIIT (*N* = 22), VLCHF (*N* = 25), VLCHF+HIIT (*N* = 25), or control (*N* = 19) groups for 12 weeks. Body composition and CRF were analyzed before the experimental period and after 4, 8, and 12 weeks. Dual-energy X-ray absorptiometry (DXA) and graded exercise test (GXT) to volitional exhaustion were used for the body composition and CRF assessments, respectively.

**Results:** There were significant between-group differences in the VAT mass and body composition outcome changes. VAT mass decreased after 12 weeks only in the VLCHF and VLCHF+HIIT groups (*p* < 0.001, median [95% CI]: VLCHF: −142.0 [−187.0; −109.5] g; VLCHF+HIIT: −104.0 [−135.0; −71.0] g). Similarly, changes in body mass, total body fat, trunk fat mass, waist and hip circumferences were distinctly decreased in the VLCHF and VLCHF+HIIT groups, when compared to HIIT and Control groups. Total lean mass significantly decreased in the VLCHF and VLCHF+HIIT groups (−2.1 [−3.0; −1.6] kg and −2.5 [−3.6; −1.8] kg, respectively) after 12 weeks. While the HIIT program significantly increased total time to exhaustion in the GXT, peak oxygen uptake was unchanged.

**Conclusions:** A VLCHF diet, either in isolation or in combination with HIIT, was shown to induce a significant reduction in VAT mass and body composition variables. HIIT alone did not cause such effects on body composition, but improved exercise capacity. Our findings indicate that the VLCHF diet and exercise training provoked different and isolated effects on body composition and CRF.

**Clinical Trial Registration**: https://clinicaltrials.gov/ct2/show/NCT03934476, identifier: NCT03934476.

## Introduction

Obesity rates have grown to epidemic proportions globally since the 1970s, and the number of obese people worldwide now exceeds those who are underweight. However, 20–40 percent of normal-weight non-obese individuals also may have excess body fat (1). Termed overfat, excess body fat can impair fat metabolism, directly influence health and fitness, and is a global pandemic (2). Both the overfat condition and reduced fat-free (muscle) mass may lower health (3). Being overfat can promote chronic low-grade systemic inflammation (4) which contributes to reduced life expectancy, impaired quality of life, and development of cardiovascular diseases, type 2 diabetes mellitus, osteoarthritis, cancer (5), hypertension (6) and non-alcoholic fatty liver disease (7).

The prevention and treatment of the overfat condition is much more complicated than just an easy recommendation to eat less and move more. The traditional strategy of counting calories does not seem adequate for solving this global pandemic. However, nutrition is still considered one of the main factors contributing to excess body fat. Notably, a very low-carbohydrate, high-fat (VLCHF) diet has been shown to beneficially affect body composition (8, 9). For example, an 8 week carbohydrate restricted diet caused 3-fold greater loss in visceral adipose tissue (VAT) compared to a standard carbohydrate-based/low-fat diet in obese (10). One explanation is that this may be due to an increase in total energy expenditure induced by lower carbohydrate diets adhered to for at least 2.5 weeks (11). Besides the loss of excess body fat, a reduction in dietary carbohydrate intake and nutritionally induced ketosis cause beneficial changes in muscle metabolism, mitochondrial function, and efficiency (12). These changes might be an effective treatment for non-alcoholic fatty liver disease (13), diabetes (14), protection of the aging brain (15, 16), blocking NLRP3 inflammasome-mediated inflammatory diseases such as Alzheimer's disease, and reduce oxidative stress, cancer growth, angiogenesis, and atherosclerosis (17, 18).

Physical activity, combined with nutrition, psychosocial factors, and genetics, plays a fundamental role in preventing and treating excess body fat and its associated chronic diseases. These general physical activity recommendations have been described elsewhere (19). Regular exercise improves cardiorespiratory fitness (CRF) and major markers of cardiometabolic health independently to body mass loss. Exercise training-induced body mass loss, even if it is usually negligible, decreases visceral abdominal fat even more than energy restriction-induced body mass loss (20). High-intensity interval training (HIIT) is a popular exercise format for health and fitness purposes (21). HIIT improves maximal aerobic capacity (VO_2max_) and cardiometabolic health (22–24), endothelial function in overweight/obese adults (25, 26), resting blood pressure, metabolic capacity, and heart rate reserve in sedentary aging men (27), as well as attenuates oxidative stress and upregulates antioxidant capacity (28). HIIT is more effective in these outcomes than moderate-intensity continuous training and is considered safe for most individuals, even those with elevated cardiometabolic risk (27, 29). The effects of HIIT and moderate-intensity continuous training on body composition are similar but the duration of HIIT is much shorter (30). Therefore, HIIT may be a better tool for body mass management programs which need to be sustainable.

Nutrition and physical activity are, therefore, the main pillars of all recommendations for a healthy and sustainable lifestyle. Both these lifestyle interventions are effective in promoting a healthy adipose tissue phenotype and even in reversing adipose tissue dysfunction and related adverse cardiometabolic effects (31). However, the beneficial effects of both the VLCHF diet and HIIT on body composition and fitness levels have not yet been shown. Susceptibility to obesity-related cardiometabolic complications is largely dependent upon individual differences in regional body fat distribution (32). Therefore, the purpose of this study was to investigate the isolated and synergic effects of a 12 week VLCHF diet and HIIT program on visceral fat deposition and CRF in overfat individuals.

## Methods

### Participants

**Figure 1:**
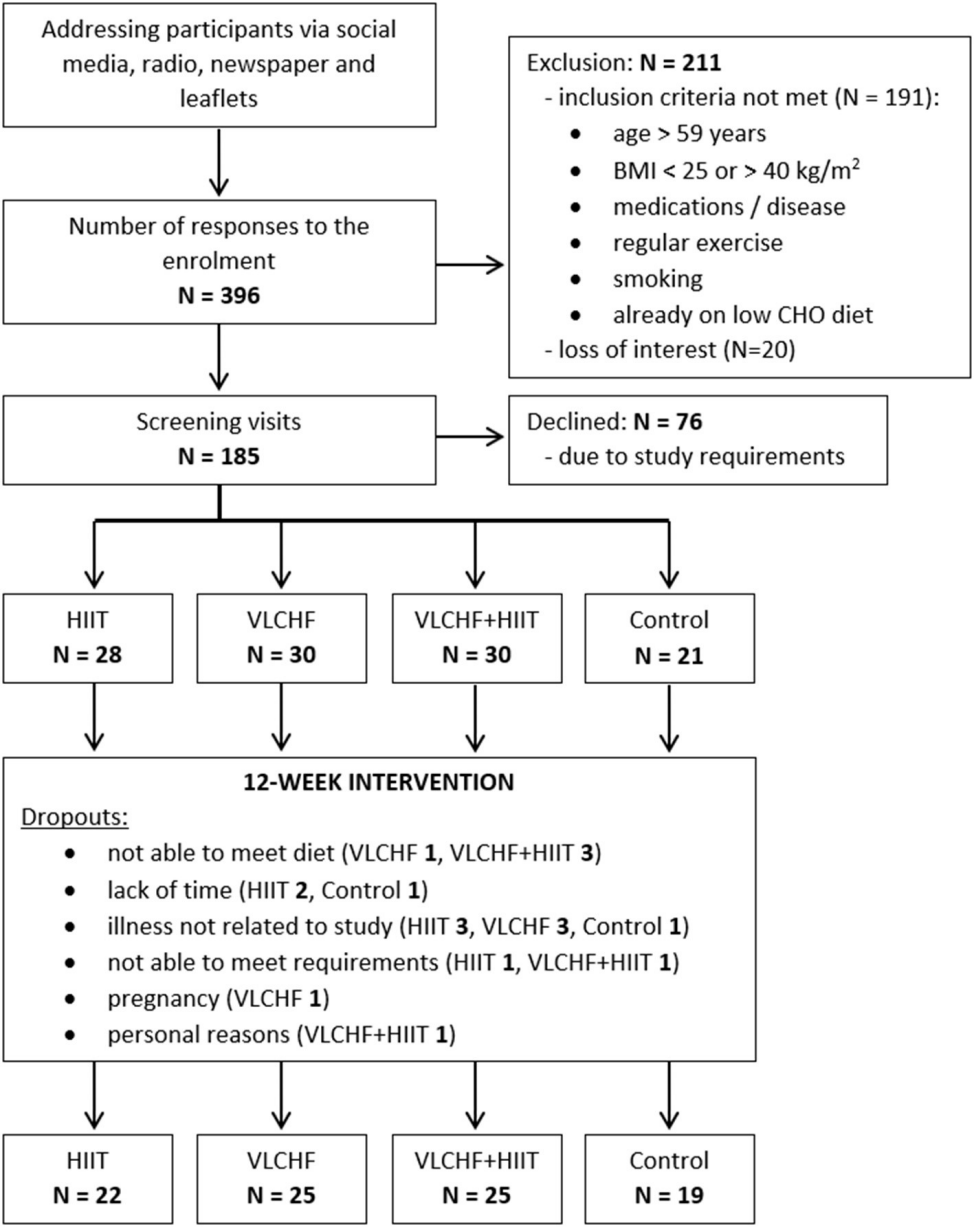
Flow chart.

Participants were randomly allocated to four study groups: (1) high-intensity interval training (HIIT) and habitual diet, (2) very low-carbohydrate, high-fat diet (VLCHF) and habitual physical activity (no regular exercise training), (3) VLCHF diet and HIIT, and (4) control (habitual diet and physical activity, no regular exercise training) (Table 1). The inclusion criteria were age 20–59 years, non-smokers, overweight/obesity (BMI 25.00–40.00 kg/m^2^), no specific sports training or regular exercise (low physical activity), no excessive alcohol intake, willingness to accept random assignment, self-reported no evidence of liver, renal, metabolic, and cardiopulmonary disease, and diseases contraindicating physical activity, no cancer, no psychiatric illness, no pregnancy or breast-feeding, no specific diet, PAR-Q pass, body mass stable for the last 2 months (<5% of total body mass), and not on a weight-loss plan, no hypoglycemic, lipid-lowering, antihypertensive, psychiatric medications, or medications known to affect body weight or energy expenditure. The participants had no previous experience with the VLCHF diet or HIIT. The recruitment details and dropouts during the study are shown in Figure 1. Written informed consent was obtained from all study participants. The study design was approved by the local University ethics committee.

**Table 1:** Baseline characteristics of study participants.

	**HIIT (*N* = 22)**	**VLCHF (*N* = 25)**	**VLCHF+HIIT (*N* = 25)**	**Control (*N* = 19)**
Male: Female	6:16	8:17	7:18	6:13
Age (year)	46 (38.8; 53.3)	43 (35.0; 51.5)	43 (32; 51)	40 (31; 53)
Height (cm)	167.7 (160.6; 175.0)	170.1 (164.7; 177.9)	169.8 (160.9; 179.7)	171.9 (162.8; 177.6)
BMI (kg.m^−2^)	28.7 (26.9; 30.9)	31.3 (27.7; 33.0)	31.0 (27.2; 35.1)	28.7 (26.7; 32.9)
WHtR (–)	0.60 (0.54; 0.62)	0.62 (0.56; 0.65)	0.62 (0.54; 0.68)	0.60 (0.54; 0.63)

*BMI, body mass index; WHtR, waist-to-height ratio*.

*Values are shown as median (lower and upper quartile)*.

### Study Design

It was a randomized, controlled, four-arm, parallel exercise and/or dietary intervention study (ClinicalTrials.gov: NCT03934476). The randomization process was stratified according to age (20–29, 30–39, 40–49, 50–59 years) and sex (male, female). The condition assignments were placed by a principal researcher in envelopes that were drawn by the participants. The study group assignments were randomly permuted within blocks of 8 participants (2 participants randomly allocated to each study arm). There were 91 participants (27 males, 64 females) allocated to the four study groups and these completed a 12 week experimental period (Figure 1). Body composition and fitness level were analyzed before the experimental period (T_0_) and after 4, 8, and 12 weeks (T_1_, T_2_, and T_3_).

A control group was included to obtain measures for no intervention. Participants in the control group were advised not to change their habitual diet and physical activity regime. No diet advice was given. Participants were tested at T_0_-T_3_.

### High-Intensity Interval Training (HIIT)

**Figure 2:**
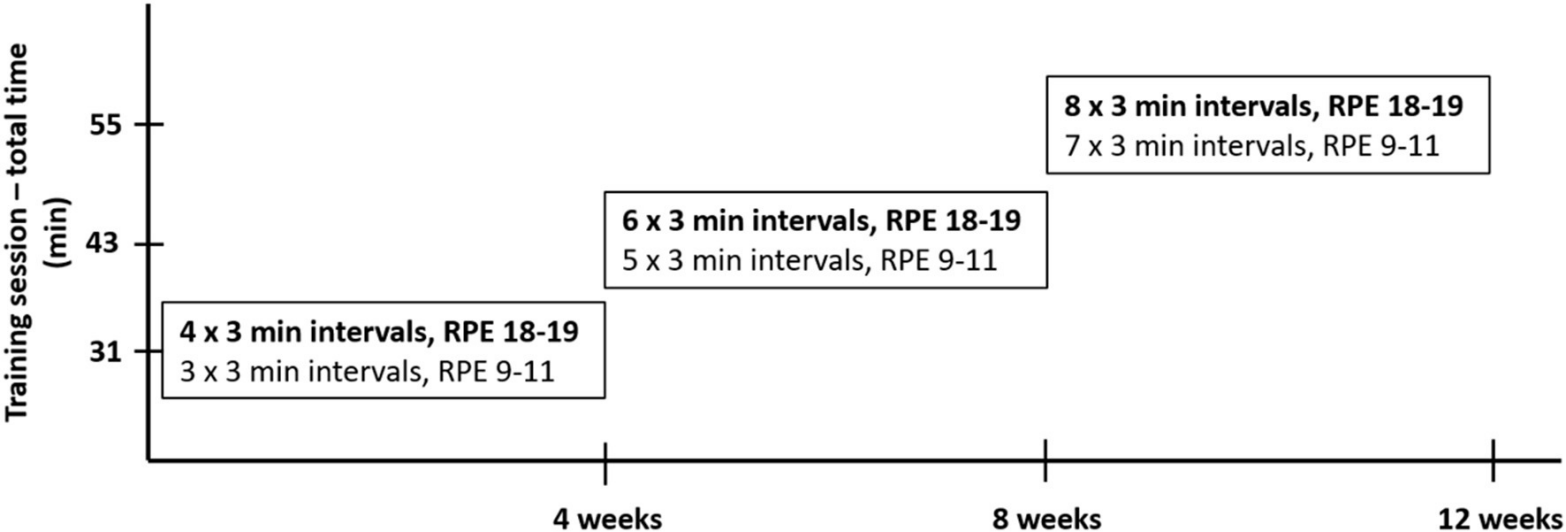
HIIT design. The 5-min warm-up and 5-min cool-down were included in every training session.

Before starting the intervention, researchers provided detailed instructions on the HIIT program to the participants in both the HIIT and VLCHF+HIIT groups in person verbally and in written form. The participants were told to complete self-performed three sessions per week. Two HIIT sessions were completed during weeks 4, 8, and 12 when the participants visited the laboratory. Each HIIT session was started and finished with 5 min of slow walking. HIIT consisted of a 3 min interval of high-intensity walking (Borg's scale RPE 18–19) followed by a 3 min interval of low-intensity walking (RPE 9–11). There were 4, 6, and 8 high-intensity intervals for the first, second, and third 4 week period. Therefore, the total session time increased from 31 to 43 min and 55 min during each 4 weeks (Figure 2). Training intensity was measured with a heart rate monitor (Polar M430; Polar Electro, Oy, Finland), and training data were uploaded to Polar Flow and analyzed regularly and to track compliance. Participants were obliged to record all additional training sessions of any type in addition to the study protocol.

### Dietary Intervention

Participants in both the HIIT and control groups were asked to maintain their habitual dietary intake without restriction. The VLCHF diet was defined as allowing no more than 50 g/day of CHO (33). Neither diet included a specific calorie or energy goal. However, participants in the VLCHF group were advised to compensate for the total energy decrease caused by CHO intake restriction by increasing their natural non-trans fat intake (e.g., cream, butter, olive, and coconut oil). A target protein intake of 1.5 g/kg lean body mass was recommended, and unlike the strict CHO restriction, participants were asked to keep to targets. The use of all sweetened and grain-based products was to be minimized. The recommended food included whole food sources, such as meats, vegetables, non-sweetened products, full-fat dairy items, nuts, and seeds. A dietitian provided detailed dietary advice before and during the study (on request or at least once a month). A handbook was given to participants containing food lists, guidelines for estimating macronutrient amounts, and sample recipes. All foods and quantities consumed were recorded daily in all study groups beginning 7 days before the intervention period (www.kaloricketabulky.cz). Alcoholic beverages were restricted during the intervention period, and dietary supplements were not permitted 1 month before and during the intervention period, while caffeinated beverages were restricted only before the laboratory sessions.

### Body Composition

Body mass and composition were determined using dual-energy X-ray absorptiometry (DXA; Hologic Discovery A, Waltham, MA, USA). The DXA measurement shows an excellent reliability for VAT assessment (ICC > 0.98) however it might underestimate longitudinal changes when compared to magnetic resonance imaging (34). Bioelectrical impedance (BI) analyzer (InBody770, Seoul, Korea) was used only for the waist and hip circumference measurements. Whole-body DXA scan and BI were performed on all participants at baseline and every 4 weeks (four times in total; T_0_-T_3_). Participants were asked to empty their bladder and bowel if possible. They wore only underwear during the evaluation and removed any metal and jewelry before assessment. Participants were fasted and were asked not to drink an excessive amount of fluids 2 h before the measurement.

### Graded Exercise Test

Before the intervention and at the start of each 4 weeks (four times in total), the participants underwent a laboratory-graded exercise test (GXT) on a motorized treadmill to determine the total time to exhaustion (TTE), peak aerobic power ($\overset{∙}{\text{V}}$O_2peak_), second ventilatory threshold (VT_2_), and respiratory exchange ratio (RER). The Balke-Ware treadmill protocol (35) was used: the inclination increased from 2.0% every minute by 1.0%, while the speed remained at 5.3 km/h until volitional exhaustion. Expired air was continuously monitored to analyze O_2_ and CO_2_ concentrations during the GXT with a breath-by-breath system (Blue Cherry, Geratherm Medical AG, Germany). The highest average O_2_ consumption measured over a 30 s period was used to determine $\overset{∙}{\text{V}}$O_2peak_. Gas exchange measurements were also used to quantify VT_2_. A second sharp increase in ventilation (VE) accompanied by an increase in VE/VO_2_ and VE/VCO_2_ was used to define VT_2_ (36). $\overset{∙}{\text{V}}$O_2peak_ can be determined with the within-subject coefficient of variation (CV) between 4 and 9% (37). Heart rate (HR) was measured using a chest belt monitor (Polar Electro H9, Kempele, Finland). Each participant performed the laboratory sessions at a similar time of the day (±60 min). All sessions were conducted at least 3 h after the participants' last meal and in a thermally controlled laboratory (21°C, 40% relative humidity). Each participant was advised not to participate in any vigorous activity 24 h before the test.

### Laboratory Methods

A capillary blood sample was drawn from a finger to measure β-hydroxybutyrate (βHB) (FreeStyle Optium Neo, Oxon, United Kingdom). Participants self-analyzed βHB twice a week (every Monday and Thursday) in a fasting state in the morning.

### Statistical Analyses

The categorical variable (sex) was described by frequency ratio, and numerical variables were described by median and interquartile range at each time point. Subsequently, the absolute changes of the monitored variables at time T_1_, T_2_, and T_3_ with respect to the baseline (T_0_) were analyzed. The absolute changes were tested for normal distribution using the Shapiro-Wilk test. In some cases, significant deviations from normality were detected such that non-parametric methods of data description (median and interquartile range) and statistical inference were used. Significance of change was tested by 95% confidence interval (CI) of median and two-tailed Wilcoxon signed-rank test for each variable, each group, and each time. The effect size (ES) of the observed changes was specified by the Wilcoxon effect size (*r*), including its 95% confidence interval. Threshold values for ES were 0.10 to < 0.30 (small), 0.30 to < 0.50 (medium), ≥ 0.50 (large).

Finally, the absolute changes in the given variables for the HIIT, VLCHF, HIIT+VLCHF, and Control groups were compared using the Kruskal-Wallis test at each time point. Dunn's test was used to analyze specific sample pairs for stochastic dominance. The effect size of the observed differences was assessed using the eta squared based on the H-statistic, including its 95% confidence interval. Threshold values for ES eta squared were 0.01 to <0.08 (small), 0.08 to <0.26 (medium), ≥0.26 (large) (38).

An a priori power analysis using GPOWER (39) with power set at 0.80 and significance level set at 0.05 was calculated retrospectively. The power analysis indicated that a total sample of 76 people would be needed to detect large effects (f = 0.40) for this study with 4 groups. A total sample of 180 people would be needed to detect medium effects (f = 0.25) (40). Thus, the sample size was sufficient to reveal that a large effect could not be interpreted as non-significant.

In all cases, statistical significance was set at *p* < 0.05. Statistical analyses were performed using R Core Team (41).

## Results

### High-Intensity Interval Training

There were substantial between-group differences in the training characteristics. Total training time in the HIIT and VLCHF+HIIT groups (median 1,424 and 1,452 min, respectively) was substantially higher than in the groups without the HIIT intervention (VLCHF-−124 min, Control 105 min). All training sessions recorded by the HR monitors are presented in Table 2. These results show all the monitored training sessions, including HIIT sessions in the HIIT and VLCHF+HIIT groups.

**Table 2:** Training sessions characteristics during the 12 week interventions.

	**HIIT**	**VLCHF**	**VLCHF+HIIT**	**Control**
TTT (min)	1,424 (1,315; 1,872)	124 (0; 302)	1,452 (1,246; 1,844)	105 (0; 653)
TS (nr)	35 (32; 41)	2 (0; 7)	33 (28; 38)	3 (0; 12)
HR_max_ (bpm)	152 (139; 159)	139 (124; 163)	152 (139; 167)	144 (124; 150)
HR_mean_ (bpm)	123 (115; 128)	113 (99; 131)	124 (113; 134)	112 (99; 117)

*TTT, total training time; TS, number of monitored training sessions; HR_max_, maximal heart rate; HR_mean_, mean heart rate*.

*Values are shown as median (lower and upper quartile)*.

### Diet

**Figure 3:**
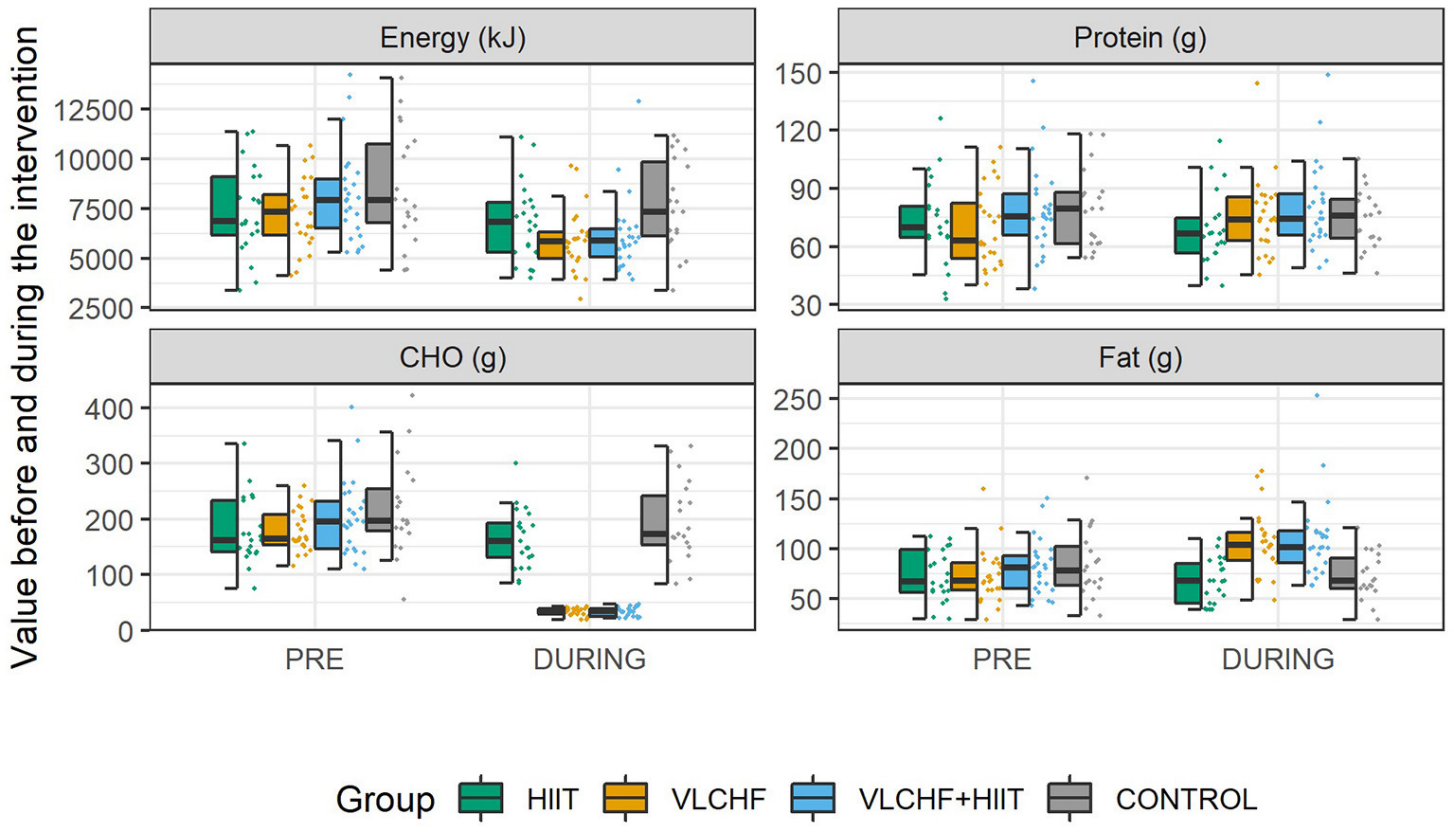
Diet characteristics before (PRE; 7-day record) and during the 12 week intervention.

Total energy intake decreased (*p* < 0.05) in the HIIT (median [95% CI]: 6.1 [0.2; 13.4] %), VLCHF (19.7 [12.5; 25.2] %), and VLCHF+HIIT (25.8 [20.5; 28.0] %) groups. Carbohydrate intake decreased (*p* < 0.05) by 81.8 [79.1; 82.9] % and 82.8 [80.4; 85.7] % in the VLCHF and VLCHF+HIIT groups, respectively. Fat intake increased by 44.6 [36.1; 61.7] % and 34.8 [24.6; 47.3] % in the VLCHF and VLCHF+HIIT groups, respectively. Protein intake did not significantly change in any of the study groups. Total energy, protein, and carbohydrate intake did not significantly change in the control group, whereas fat intake decreased (*p* = 0.023, 6.0 [1.0; 17.5] %) (Figure 3, Supplementary Tables 1, 2).

**Figure 4:**
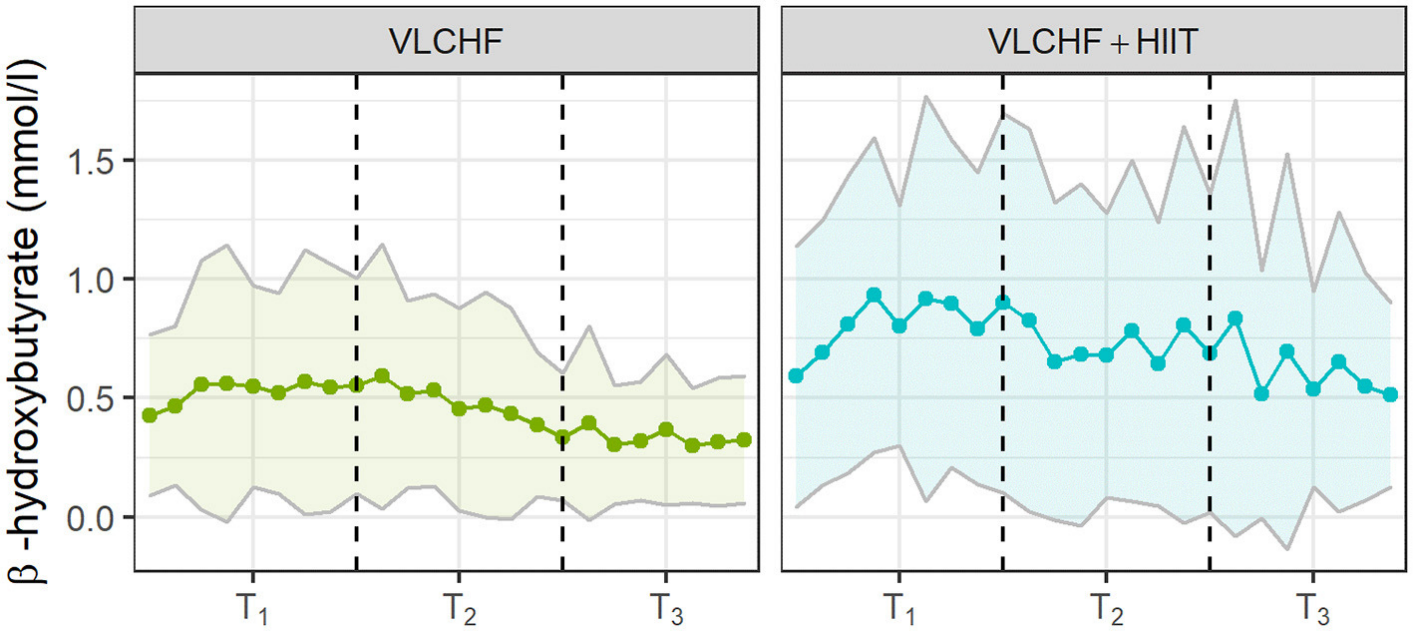
The capillary β-hydroxybutyrate concentrations in the VLCHF and VLCHF+HIIT groups. The βHB concentrations were self-analysed twice a week. The data are shown as mean and SD.

The β-hydroxybutyrate concentration (βHB) increased substantially in the VLCHF and VLCHF+HIIT groups. The highest βHB concentrations were achieved after 2 weeks of VLCHF diet intervention (Figure 4). βHB concentrations in the HIIT and control groups remained within the range between 0.0 to 0.3 mmol/l for the whole 12 week intervention (data not shown).

### Body Composition

**Figure 5:**
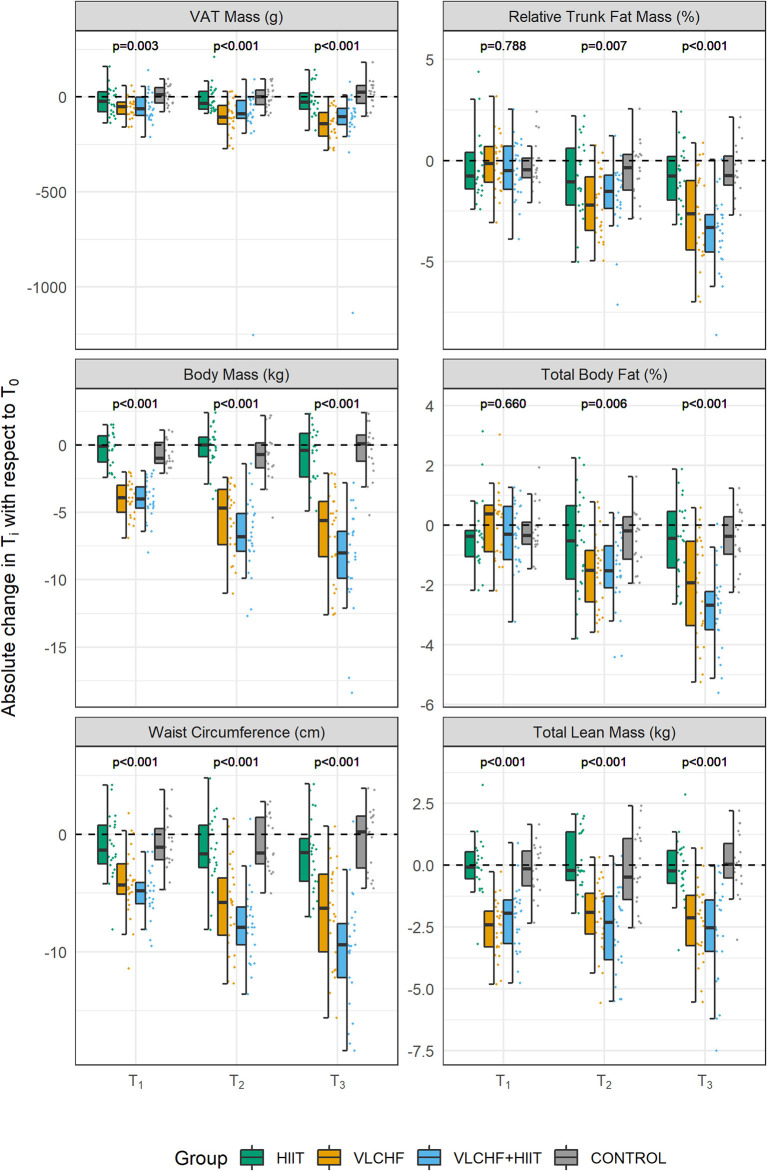
Changes in body composition variables after 4, 8, and 12 weeks (T_1_, T_2_, T_3_, respectively). VAT, visceral adipose tissue. *p*-values—Kruskal-Wallis test for the between-group differences. *Post-hoc* test results are shown in the Table 3.

There were large between-group differences in visceral adipose tissue (VAT) mass, area and volume changes after 12 weeks (*p* < 0.001 for all; ES [95% CI]: 0.42 [0.27; 0.58], 0.41 [0.27; 0.58], and 0.42 [0.27; 0.59], respectively). *Post-hoc* analysis revealed that 12 week VLCHF diet intervention, regardless of combination with HIIT, was effective in decreasing VAT when compared to the HIIT and Control groups (Table 3, Figure 5). The absolute VAT mass (g) reduced by 23.2 % (median; 95% CI: [25.9; 17.0] %) in the VLCHF group and 17.6 % [23.9; 12.0] in the VLCHF+HIIT group while these changes were not significant after 12 weeks in both the HIIT and Control groups. The complete dataset is reported in Supplementary Tables 3, 6. The significant VAT decrease in the VLCHF and VLCHF+HIIT groups was also revealed after 4 and 8 weeks (Supplementary Tables 4, 5, 7, 8, Figure 5).

**Table 3:** Body composition and fitness level differences after 12 weeks.

	**HIIT**	**VLCHF**	**VLCHF+HIIT**	**Control**	**Between-group diff. (*p*-value)**
	**Δ*M* (95% CI)**	**Δ*M* (95% CI)**	**Δ*M* (95% CI)**	**Δ*M* (95% CI)**	
VAT Mass (g)	−27.0 (−52.5; 27.5)	−142.0 (−187.0; −109.5)^**^	−104 (−135; −71)^**^	26 (−25; 48.5)	<0.001^a^
VAT Area (cm^2^)	−5.8 (−11.0; 5.8)	−29.5 (−39.0; −22.5)^**^	−20.9 (−25.7; −13.7)^**^	5.4 (−5.5; 10.3)	<0.001^a^
VAT Volume (cm^3^)	−29.5 (−56.5; 30.0)	−153 (−202; −118)^**^	−109 (−134.5; −72)^**^	28 (−26.5; 53)	<0.001^a^
Body mass (kg)	−0.4 (−1.6; 0.3)	−5.6 (−8.1; −4.9)^**^	−8.0 (−9.6; −6.8)^**^	0.1 (−1.3; 0.7)	<0.001^a^
Total body Fat (%)	−0.4 (−1.2; 0.1)	−1.9 (−2.9; −1.3)^**^	−2.7 (−3.4; −2.3)^**^	−0.4 (−0.9; 0.0)	<0.001^a^
Trunk fat Mass (kg)	−0.8 (−1.1; −0.1)^*^	−2.5 (−3.3; −2.2)^**^	−3.3 (−4.0; −2.8)^**^	−0.2 (−0.5; 0.3)	<0.001^a^
Relative trunk fat mass (%)	−0.8 (−1.6; −0.1)^*^	−2.6 (−3.7; −1.8)^**^	−3.3 (−4.2; −2.8)^**^	−0.7 (−1.1; 0.1)	<0.001^a^
Total lean mass (kg)	−0.2 (−0.6; 0.4)	−2.1 (−3.0; −1.6)^**^	−2.5 (−3.6; −1.8)^**^	0.0 (−0.5; 0.7)	<0.001^a^
Waist circumference (cm)	−1.5 (−3.3; −0.3)^*^	−6.3 (−8.6; −4.7)^**^	−9.4 (−11.8; −7.9)^**^	0.2 (−1.6; 1.3)	<0.001^a^
Hip circumference (cm)	−0.5 (−0.8; 0.1)	−3.4 (−4.2; −2.6)^**^	−3.7 (−4.8; −3.4)^**^	−0.2 (−0.9; 0.3)	<0.001^a^
WHR (–)	−0.021 (−0.030; −0.001)^*^	−0.027 (−0.046; −0.019)^**^	−0.053 (−0.069; −0.041)^**^	0.005 (−0.011; 0.013)	<0.001^b^
WHtR (–)	−0.009 (−0.019; −0.002)^*^	−0.038 (−0.049; −0.028)^**^	−0.053 (−0.069; −0.047)^**^	0.001 (−0.009; 0.008)	<0.001^a^
TTE (min:s)	1:35 (0:46; 2:07)^**^	0:48 (0:07; 1:21)^*^	1:49 (1:02; 2:17)^**^	0:02 (−0:24; 0:51)	0.004^c^
VO_2peak_ (l/min)	0.10 (−0.05; 0.32)	−0.07 (−0.14; 0.06)	0.04 (−0.14; 0.15)	−0.11 (−0.29; 0.02)	0.137
VO_2peak_ (ml/kg/min)	1.45 (−0.25; 3.65)	0.90 (0.10; 2.55)^*^	3.45 (1.25; 4.20)^*^	−1.20 (−3.05; 0.45)	0.008^d^
RER_peak_	−0.01 (−0.04; 0.04)	−0.04 (−0.08; −0.02)^*^	−0.07 (−0.11; −0.02)^*^	0.03 (0.00; 0.05)^*^	0.002^b^
HR_peak_ (bpm)	−1.0 (−5.0; 2.5)	1.0 (−3.0; 3.5)	0.5 (−3.5; 3.0)	−3.0 (−7.0; −0.5)^*^	0.346
VT_2_ (% VO_2peak_)	1.0 (−1.5; 4.0)	0.0 (−2.0; 2.5)	−1.0 (−4.5; 0.5)	1.0 (−2.5; 4.5)	0.314

*VAT, visceral adipose tissue; WHR, waist-to-hip ratio; WHtR, waist-to-height ratio; TTE, total time to exhaustion; VO_2peak_, peak oxygen consumption; RER_peak_, peak respiratory exchange ratio; HR_peak_, peak heart rate; VT_2_, second ventilatory threshold*.

*Data are the median differences (ΔM) between baseline minus 12 week measures with 95% confidence intervals (CI). The complete dataset is reported in the Supplementary Material*.

*Two-tailed Wilcoxon signed-rank test:*

* *Significant differences (p < 0.05) for baseline vs. 12 week;*

** *Significant differences (p < 0.001) for baseline vs. 12 week*.

*Kruskal-Wallis test for the between-group differences. Post-hoc analysis:*

a *–VLCHF and VLCHF+HIIT vs. HIIT and Control*,

b *–HIIT and Control vs. HIIT and VLCHF vs. VLCHF+HIIT*,

c *–HIIT and VLCHF vs. VLCHF and Control vs. VLCHF+HIIT vs. Control, and*

d *–HIIT and VLCHF and VLCHF+HIIT vs. Control*.

Total lean mass significantly decreased after 4 weeks by 4.7 [3.9; 5.7] % and 3.9 [3.1; 5.1] % in the VLCHF and VLCHF+HIIT groups, respectively. These changes remained stable over the 8 and 12 week measurements (4.2 [3.1; 5.2] % and 4.9 [3.8; 6.5] %, respectively) (Figure 5). Significant between-group differences in all other monitored body composition variables, with the most pronounced changes in the VLCHF and VLCHF+HIIT groups, were shown after 12 weeks (Table 3, Figure 5).

### Graded Exercise Test

**Figure 6:**
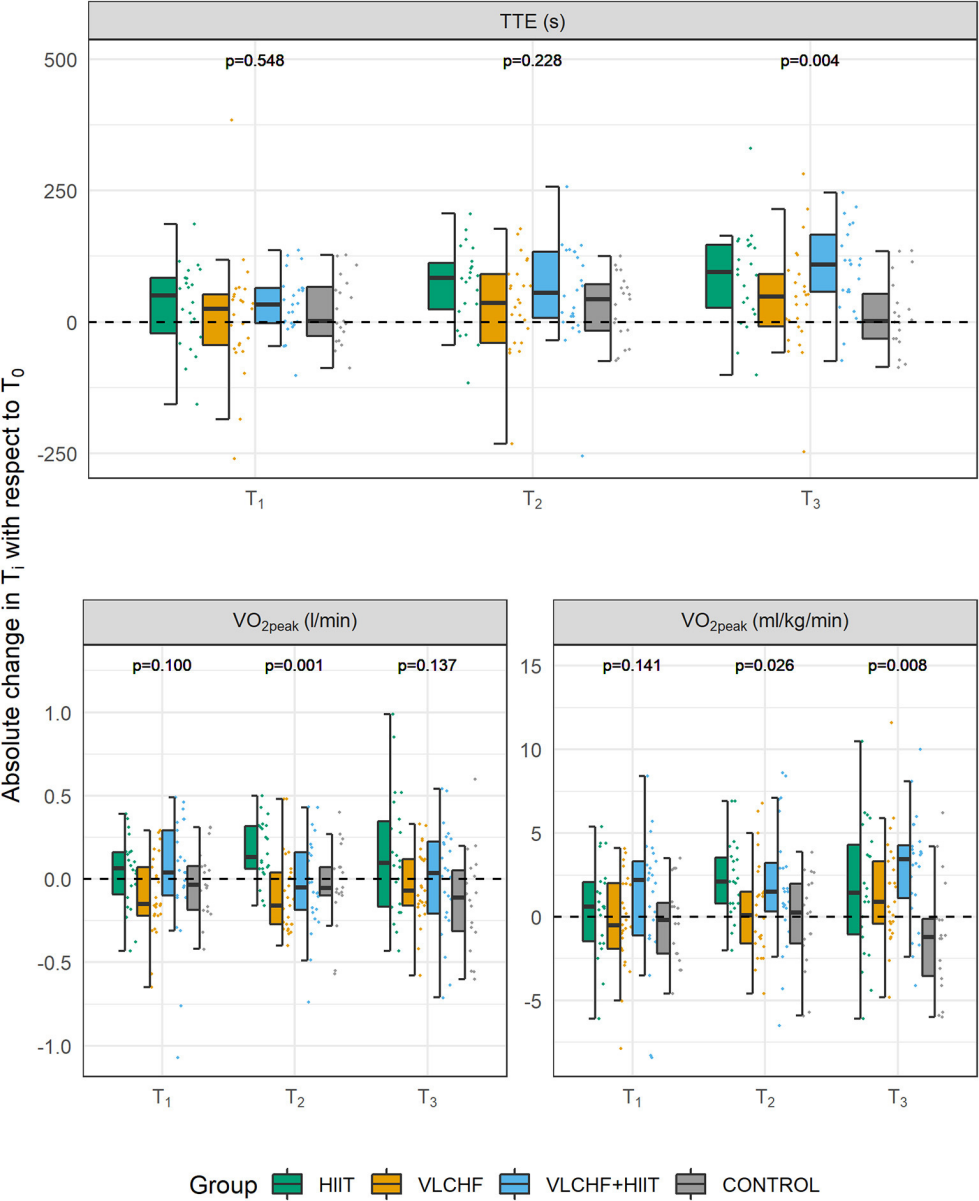
Changes in GXT variables after 4, 8, and 12 weeks (T_1_, T_2_, T_3_, respectively). TTE, total time to exhaustion; $\overset{∙}{\text{V}}$O_2peak_, peak oxygen consumption. *p*-values—Kruskal-Wallis test for the between-group differences. *Post-hoc* test results are shown in the Table 3.

There were small to large between-group differences in TTE and relative $\overset{∙}{\text{V}}$O_2peak_ changes after 12 weeks (*p* < 0.05 for both; ES [95% CI]: 0.15 [0.06; 0.33] and 0.13 [0.04; 0.31], respectively). *Post-hoc* analysis revealed TTE differences between HIIT and VLCHF+HIIT vs. VLCHF and Control groups. TTE increased after 12 weeks significantly by 12.0% (median; 95% CI [5.8; 17.4] %), 16.9 [10.1; 24.7] %, and 5.9 [1.0; 13.3] % in the HIIT, VLCHF+HIIT, and VLCHF diet groups, respectively. The relative $\overset{∙}{\text{V}}$O_2peak_ was significantly different in all intervention groups compared to the Control group after 12 weeks. The relative $\overset{∙}{\text{V}}$O_2peak_ increased after 12 weeks by 4.9 [−1.6; 12.3] %, 11.9 [3.9; 15.9] %, and 3.7 [0.6; 9.6] % in the HIIT, VLCHF+HIIT, and VLCHF groups, respectively. Small to large between group differences after 12 weeks were found also in the RER_peak_ (*p* = 0.002; 0.17 [0.07; 0.35]). *Post-hoc* analysis showed significantly lower RER_peak_ in the VLCHF and VLCHF+HIIT groups when compared to the HIIT and Control groups (Table 3, Figure 6). The complete dataset, including the outcomes after 4 and 8 weeks, is reported in Supplementary Tables 3–6. VT_2_ was not significantly different between all study groups after 4, 8, and 12 weeks (Table 3, Supplementary Tables 7, 8).

## Discussion

The aim of this randomized controlled study was to investigate whether a very low-carbohydrate high-fat (VLCHF) diet and high-intensity interval training (HIIT) program worked in isolation or synergically to effect VAT and CRF. The findings showed that VAT was significantly decreased in the VLCHF and VLCHF+HIIT groups. HIIT alone did not cause a substantial VAT decrease, nor did it show any significant changes in other body composition variables compared to the Control group. When HIIT was combined with the VLCHF diet, no extra VAT changes were revealed either. However, HIIT alone, as well as in combination with the VLCHF diet, substantially improved exercise capacity. These results showed that a VLCHF diet had a greater effect on body composition management than exercise alone. However, a slight decrease of lean body mass in both diet groups needs to be highlighted.

### VLCHF Diet and βHB

A reduced CHO diet is characterized by a CHO intake below 45% of the total energy intake. The VLCHF diet intervention in this study required a CHO intake of <50 g/day (14). Since the purpose of the VLCHF diet intervention in this study was not to reduce the total energy intake, the participants in the VLCHF and VLCHF+HIIT groups were encouraged to compensate for the CHO intake restriction by increasing fat intake while maintaining protein consumption. Nevertheless, fat intake was not sufficient to keep the total energy intake unchanged (Figure 3), which is a common situation in real-life conditions. Notably, total energy intake significantly decreased in the HIIT group despite there being no diet modification. We attribute this effect to an increased interest in a healthy lifestyle when participating in such a research study.

As expected, the βHB concentration increased significantly with CHO restriction, although nutritional ketosis was not primarily intended (Figure 4). These βHB changes in both diet groups are in agreement with our previous results (42, 43) and have also been shown previously (13, 44, 45). However, the pattern of βHB changes was different between the VLCHF and VLCHF+HIIT groups. Higher and more variable βHB concentrations were found in the VLCHF+HIIT group. This was likely caused by glycogen depletion due to regular high-intensity exercise sessions, which increased nutritional ketosis. We assume that the higher βHB fluctuation corresponds to temporary muscle glycogen store depletion in response to HIIT and reduced endogenous glucose synthesis during the early phase of VLCHF diet intervention. Indeed, muscle glycogen stores are not diminished in VLCHF diet-adapted individuals (46). Our study results are unique as they show that nutritional ketosis (if intended) can be supported by high-intensity exercise and not only provoked by strict CHO intake manipulation.

Interestingly, βHB tended to decrease toward the end of the 12 week intervention period, which is in agreement with our previous results (42). This may reflect enhanced cellular uptake after adaptation as a result of enhanced mitochondrial function and capacity for ketone body utilization (47). Another possible explanation for this trend is an increase in gluconeogenetic glucose production (48).

### Body Composition

The VLCHF diet, both with and without HIIT, caused significant changes in body composition of these overfat individuals. We found large (ES) between-group differences in the VAT mass changes, which significantly decreased in the VLCHF (by ~23.2) and VLCHF+HIIT (by ~17.6 %) groups (Figure 5). A more substantial reduction in central adiposity was also observed in obese adults when a low-CHO diet was compared to a low-fat diet (9, 49, 50). A low-CHO diet is considered a legitimate and potentially effective form of treatment for patients with obesity (51). A possible explanation for the carbohydrate-restricted diet superiority over a low-fat diet on body composition might be the increasing long-term effect on total energy expenditure (11). However, we cannot relate the presented body composition changes unequivocally to the VLCHF diet intervention because the total energy intake in the VLCHF and VLCHF+HIIT groups was also significantly decreased. On the other hand, the total energy intake significantly decreased in the HIIT group, but without a pronounced effect on body mass and composition.

The total lean mass significantly decreased abruptly in the VLCHF and VLCHF+HIIT groups after 4 weeks, but then remained relatively stable. This can be explained by both the sharp reduction in total energy intake or the macronutrient shift at the beginning of the VLCHF diet intervention. Interestingly, exercise in the VLCHF+HIIT group did not prevent this lean body mass change. Such a decrease in lean body mass, induced by a CHO restricted diet, was previously observed in obese (10) and athletic subjects (52). Although a muscle/liver glycogen reduction and following higher rate of gluconeogenesis from muscle amino acids during exercise may be suggested as a cause of the lean body mass decrease, our data do not support such an assumption since the lean body mass decrease was found similarly in the both VLCHF and VLCHF+HIIT groups. However, we have to consider that lean body mass results gained by DXA can be influenced by hydration status, i.e., DXA interprets body water loss as total lean mass loss (53). Very low CHO diets can be accompanied by body water loss due to accelerated sodium and water excretion and glycogen depletion which may cause also an additional body water loss (10). Although lean body mass decrease ceased after 4 weeks, VAT mass/area/volume continued to decrease progressively throughout the 12 week VLCHF diet intervention in the present study.

### Cardiorespiratory Fitness Level

The most pronounced TTE increase was revealed in the VLCHF+HIIT group after 12 weeks. Despite the fact that TTE increased also in the HIIT and VLCHF groups, the absolute $\overset{∙}{\text{V}}$O_2peak_ values did not change in any study group. Therefore, we attribute the significant increase of relative $\overset{∙}{\text{V}}$O_2peak_ (ml/kg/min) values in the VLCHF and VLCHF+HIIT groups rather to the rapid body mass reduction than any enhancement of aerobic capacity *per se*. Although carbohydrate restricted diets combined with HIIT were shown to enhance aerobic capacity in various populations (54) as well as in the obese (55), this rapid body mass reduction effect on relative $\overset{∙}{\text{V}}$O_2peak_ values is not usually considered (56). The walking HIIT protocol and/or 12 week intervention period were probably not sufficient enough to increase aerobic capacity in the present study, although the volume of exercise at high intensity was significantly increased in both HIIT and VLCHF+HIIT groups. However, we showed that regular HIIT sessions over 12 weeks had an obvious beneficial effect on exercise capacity enhancement (i.e., TTE). The increases in physical activity and/or cardiorespiratory fitness can be associated with greater reductions in mortality risk than is intentional body mass loss (20). Therefore, we can still consider HIIT an effective and time saving (30) tool in the prevention and treatment of chronic diseases in overfat individuals, even if the additional effect of the VLCHF diet and HIIT combination on cardiorespiratory fitness was not revealed.

RER_peak_ significantly decreased in the VLCHF and VLCHF+HIIT groups, as was shown in our previous studies (42, 43). This indicates an increased rate of fat oxidation, which has previously been shown to be potentially associated with impaired high-intensity performance because of a reduction in maximal CHO utilization (57). However, no such exercise capacity reduction was demonstrated in this study. Regarding submaximal exercise, the TTE may vary between individuals despite the lower RER and no significant changes in $\overset{∙}{\text{V}}$O_2max_ following a 4 week eucaloric ketogenic diet (58). Shaw et al. (59) showed that keto-adapted participants with RER <1.0 at $\overset{∙}{\text{V}}$O_2max_, reduced the TTE in submaximal exercise, while those with RER_max_ >1.0 preserved the TTE. We cannot confirm these results because the TTE significantly increased in the VLCHF and VLCHF+HIIT groups after 12 weeks (Figure 6). These differences might be explained by the fact that the duration of the VLCHF diet interventions, which is only 3–4 weeks in the aforementioned studies (57, 59), does not allow adequate adaptation to the VLCHF diet by increasing endogenous glucose production (60, 61).

### Limitations

Our study has some limitations. First, this real-life study did not allow us to directly collect some of the data (e.g., daily records of nutrition and exercise sessions). Therefore, the participant's adherence to all study requirements cannot be fully controlled. Second, the study groups were not balanced for sex and, despite the randomization, median age of the HIIT group was slightly higher than in the other study groups. Third, the menstrual cycle and menopause status were not considered within the data collection and analysis.

## Conclusions

The present study showed that a VLCHF diet can be an effective strategy for reducing excess body fat. The incorporated HIIT program had no additional effects on VAT and body composition variables. Our study indicates that the VLCHF diet intervention had a greater effect on body composition management than exercise alone. The HIIT program did not improve peak aerobic capacity but had additional benefits on exercise capacity outcomes. A slight lean body mass loss was revealed shortly after the VLCHF diet interventions started, and HIIT did not prevent it. The combination of VLCHF+HIIT program over 12 weeks may beneficially influence body composition and CRF.

## Data Availability Statement

The raw data supporting the conclusions of this article will be made available by the authors, without undue reservation.

## Ethics Statement

The studies involving human participants were reviewed and approved by Ostrava University Ethics Committee. The patients/participants provided their written informed consent to participate in this study.

## Author Contributions

LC and TD designed the study, collected, analyzed, interpreted the data, drafted, revised, and submitted the manuscript. ML analyzed and interpreted data and drafted the manuscript. PM interpreted data and revised the manuscript. PH and PL designed the study, interpreted the data, and drafted and revised the manuscript. All authors approved the final version of the manuscript.

## Funding

This work was supported by the Czech Science Foundation (Grant 18-08358S). The Healthy Aging in Industrial Environment project (registration number CZ.02.1.01/0.0/0.0/16_019/0000798, founded by the European Union via the Ministry of Education, Youth and Sports of the Czech Republic) supported the technical part of this research study. Statistical analysis was supported by Grant of SGS No. SP2021/103, VŠB - Technical University of Ostrava, Czech Republic.

## Conflict of Interest

The authors declare that the research was conducted in the absence of any commercial or financial relationships that could be construed as a potential conflict of interest.

## Publisher's Note

All claims expressed in this article are solely those of the authors and do not necessarily represent those of their affiliated organizations, or those of the publisher, the editors and the reviewers. Any product that may be evaluated in this article, or claim that may be made by its manufacturer, is not guaranteed or endorsed by the publisher.
