# Supplementary material for: Effects of a Very Low-Carbohydrate High-Fat Diet and High-Intensity Interval Training on Visceral Fat Deposition and Cardiorespiratory Fitness in Overfat Individuals: A Randomized Controlled Clinical Trial
Source: Front Nutr. 2021 Dec 21;8:785694. doi: 10.3389/fnut.2021.785694 (PMC8724307; doi:10.3389/fnut.2021.785694)
Supplement: Supplementary file 1 [file Table_1.docx]

***Supplementary Material***

**Table S1.** **Diet characteristics before (7-day record) and during the 12-week intervention.**

|  | **PRE** | | | | **DURING** | | | |
| --- | --- | --- | --- | --- | --- | --- | --- | --- |
|  | **HIIT** | **VLCHF** | **VLCHF+HIIT** | **Control** | **HIIT** | **VLCHF** | **VLCHF+HIIT** | **Control** |
| Energy (kJ) | 6900 (6000; 9100) | 7300 (6000; 8400) | 7900 (6300; 9100) | 7900 (6600; 10900) | 6800 (5300; 7900) | 5900 (4900; 6400) | 5900 (5100; 6500) | 7400 (5900; 10100) |
| Protein (g) | 70 (65; 86) | 63 (53; 86) | 75 (65; 88) | 79 (61; 88) | 67 (56; 76) | 74 (63; 86) | 74 (66; 89) | 76 (64; 85) |
| CHO (g) | 162 (140; 236) | 165 (154; 211) | 196 (146; 239) | 198 (175; 270) | 161 (122; 201) | 35 (28; 39) | 34 (25; 39) | 173 (150; 254) |
| Fat (g) | 67 (57; 99) | 68 (59; 86) | 81 (60; 95) | 78 (61; 106) | 68 (45; 87) | 104 (87; 118) | 101 (83; 118) | 68 (61; 94) |
| SFA (g) | 23 (17; 30) | 23 (18; 30) | 23 (18; 29) | 21 (18; 30) | 20 (14; 25) | 40 (31; 49) | 39 (31; 48) | 22 (17; 31) |
| Fiber (g) | 13 (10; 17) | 12 (10; 18) | 16 (13; 20) | 16 (11; 22) | 13 (9; 15) | 9 (7; 12) | 10 (8; 14) | 14 (11; 16) |

Legend: CHO – carbohydrates, SFA – saturated fatty acids.

Values are shown as median (lower and upper quartile).

**Table S2. Statistical inference for the dietary intake changes to the baseline levels.**

|  | **HIIT** | | **VLCHF** | | **VLCHF+HIIT** | | **Control** | |
| --- | --- | --- | --- | --- | --- | --- | --- | --- |
|  | **ES (95% CI)** | ***p*-value** | **ES (95% CI)** | ***p*-value** | **ES (95% CI)** | ***p*-value** | **ES (95% CI)** | ***p*-value** |
| Energy (kJ) | -0.45 (-0.80; -0.06) | 0.040 | -0.78 (-0.86; -0.62) | < 0.001 | -0.84 (-0.88; -0.73) | < 0.001 | -0.43 (-0.76; 0.00) | 0.060 |
| Protein (g) | -0.22 (-0.68; 0.23) | 0.330 | 0.39 (0.02; 0.67) | 0.053 | 0.13 (-0.27; 0.49) | 0.542 | -0.27 (-0.70; 0.20) | 0.258 |
| CHO (g) | -0.41 (-0.77; -0.05) | 0.065 | -0.86 (-0.88; -0.82) | < 0.001 | -0.87 (-0.88; -0.87) | < 0.001 | -0.28 (-0.67; 0.18) | 0.241 |
| Fat (g) | -0.29 (-0.69; 0.13) | 0.187 | 0.85 (0.80; 0.88) | < 0.001 | 0.82 (0.67; 0.88) | < 0.001 | 0.52 (-0.79; -0.11) | 0.023 |
| SFA (g) | -0.12 (-0.55; 0.32) | 0.602 | 0.85 (0.78; 0.88) | < 0.001 | 0.87 (0.87; 0.88) | < 0.001 | -0.36 (-0.75; 0.06) | 0.123 |
| Fiber (g) | -0.22 (-0.59; 0.22) | 0.313 | -0.72 (-0.86; -0.47) | < 0.001 | -0.72 (-0.88; -0.47) | < 0.001 | 0.15 (-0.36; 0.54) | 0.541 |

Legend*:* ES – Effect Size, CI – confidence interval, CHO – carbohydrates, SFA – saturated fatty acids.

**Table S3. Baseline body composition and graded exercise test outcomes.**

|  | **HIIT** | **VLCHF** | **VLCHF+HIIT** | **Control** |
| --- | --- | --- | --- | --- |
| VAT Mass (g) | 580 (460; 840) | 580 (510; 910) | 610 (410; 780) | 570 (450; 740) |
| VAT Area (cm^2^) | 121 (96; 174) | 120 (106; 190) | 125 (85; 163) | 119 (92; 154) |
| VAT Volume (cm^3^) | 630 (500; 910) | 630 (550; 990) | 650 (440; 850) | 620 (480; 800) |
| Body mass (kg) | 78 (75; 93) | 90 (76; 103) | 91 (71; 109) | 86 (77; 97) |
| Total Body Fat (%) | 40.2 (35.3; 42.5) | 41.8 (34.3; 44.9) | 40 (35.9; 44.9) | 38 (32.2; 42.8) |
| Trunk Fat Mass (kg) | 15.3 (12.7; 18.6) | 19 (13.7; 20.9) | 16.7 (12.6; 23) | 16.7 (13.2; 18.9) |
| Relative Trunk Fat Mass (%) | 38.3 (34.5; 42.4) | 39.1 (33.7; 43.8) | 39.1 (34.7; 44.2) | 36.9 (33.1; 41.2) |
| Total Lean Mass (kg) | 45 (43; 54) | 51 (45; 64) | 49 (42; 62) | 50 (43; 59) |
| Waist Circumference (cm) | 99 (93; 105) | 105 (94; 112) | 104 (92; 119) | 101 (90; 111) |
| Hip Circumference (cm) | 103.4 (100.6; 108.8) | 107 (102.4; 113.1) | 105.4 (101.2; 116.4) | 105.4 (100.8; 112.1) |
| WHR (-) | 0.96 (0.93; 0.99) | 0.97 (0.92; 1.01) | 0.95 (0.90; 1.03) | 0.97 (0.95; 1.00) |
| WHtR (-) | 0.60 (0.54; 0.62) | 0.62 (0.56; 0.65) | 0.62 (0.54; 0.68) | 0.60 (0.54; 0.63) |
|  |  |  |  |  |
| TTE (min:s) | 13:02 (10:04; 14:07) | 11:55 (8:26; 14:42) | 11:04 (9:03; 15:14) | 11:41 (11:00; 17:04) |
| VO_2peak_ (l/min) | 2.43 (2.15; 2.92) | 2.53 (2.13; 3.13) | 2.42 (1.93; 3.27) | 2.68 (2.39; 3.84) |
| VO_2peak_ (ml/kg/min) | 28.75 (26.78; 32.63) | 29.70 (22.90; 34.90) | 29.50 (24.40; 36.40) | 29.70 (27.78; 39.28) |
| RER_peak_ | 1.04 (0.98; 1.10) | 1.01 (0.97; 1.06) | 1.02 (0.93; 1.10) | 1.02 (0.97; 1.08) |
| HR_peak_ (bpm) | 181.5 (167.8; 188.3) | 179.5 (163.8; 191.8) | 181.0 (168.5; 187.0) | 181.0 (173.0; 190.0) |
| VT_2_ (% VO_2peak_) | 89.0 (81.8; 92.3) | 89.0 (86.0; 92.0) | 92.0 (87.5; 95.0) | 88.0 (84.0; 93.0) |

Legend*:* VAT – visceral adipose tissue, WHR – Waist-to-Hip Ratio, WHtR – Waist-to-Height Ratio, TTE – total time to exhaustion, VO_2peak_ – peak oxygen consumption, RER_peak_ – peak respiratory exchange ratio, HR_peak_ – peak heart rate, VT_2_ – second ventilatory threshold.

Values are shown as median (lower and upper quartile).

**Table S4. 4-week body composition and graded exercise test outcomes.**

|  | **HIIT** | **VLCHF** | **VLCHF+HIIT** | **Control** |
| --- | --- | --- | --- | --- |
| VAT Mass (g) | 580 (450; 780) | 560 (450; 850) | 560 (330; 730) | 590 (440; 830) |
| VAT Area (cm^2^) | 120 (94; 161) | 115 (93; 178) | 117 (68; 151) | 121 (91; 172) |
| VAT Volume (cm^3^) | 630 (490; 840) | 600 (490; 920) | 610 (350; 790) | 630 (470; 890) |
| Body mass (kg) | 77 (74; 94) | 87 (73; 99) | 88 (68; 104) | 86 (76; 96) |
| Total Body Fat (%) | 39.8 (35.4; 43.1) | 40.4 (33.7; 45.4) | 39.6 (35.9; 44) | 37.9 (32; 42.5) |
| Trunk Fat Mass (kg) | 15.9 (12.4; 18.1) | 17.1 (12.8; 20) | 17 (11.5; 21.3) | 16.8 (13; 18.9) |
| Relative Trunk Fat Mass (%) | 38.4 (34.1; 41.6) | 38.5 (34.1; 44.2) | 38.2 (33.3; 43) | 37.6 (32.8; 39.8) |
| Total Lean Mass (kg) | 45 (42; 57) | 49 (43; 60) | 49 (40; 59) | 50 (44; 58) |
| Waist Circumference (cm) | 98 (93; 105) | 101 (90; 109) | 99 (87; 114) | 103 (90; 109) |
| Hip Circumference (cm) | 102.9 (101.3; 109.3) | 105 (101.3; 111.8) | 104.1 (99.8; 114.4) | 105.3 (100.5; 111.7) |
| WHR (-) | 1.0 (0.9; 1.0) | 1.0 (0.9; 1.0) | 0.9 (0.9; 1.0) | 1.0 (0.9; 1.0) |
| WHtR (-) | 0.6 (0.5; 0.6) | 0.6 (0.5; 0.6) | 0.6 (0.5; 0.6) | 0.6 (0.5; 0.6) |
|  |  |  |  |  |
| TTE (min:s) | 13:02 (10:44; 15:17) | 12:43 (8:35; 15:04) | 12:01 (9:16; 15:28) | 13:07 (10:49; 16:47) |
| VO_2peak_ (l/min) | 2.42 (2.16; 3.04) | 2.36 (2.03; 3.15) | 2.53 (2.03; 3.39) | 2.73 (2.25; 3.39) |
| VO_2peak_ (ml/kg/min) | 29.95 (28.10; 33.40) | 30.20 (22.25; 34.65) | 32.10 (25.85; 35.40) | 31.40 (26.40; 35.40) |
| RER_peak_ | 1.02 (0.95; 1.05) | 0.94 (0.90; 1.00) | 0.95 (0.91; 0.98) | 0.99 (0.95; 1.05) |
| HR_peak_ (bpm) | 176.0 (168.0; 185.3) | 184.0 (166.0; 191.0) | 183.0 (174.5; 189.5) | 181.0 (172.0; 188.0) |
| VT_2_ (% VO_2peak_) | 90.5 (85.0; 93.3) | 90.0 (86.0; 92.0) | 91.0 (88.0; 95.0) | 89.0 (84.0; 91.0) |

Legend*:* VAT – visceral adipose tissue, WHR – Waist-to-Hip Ratio, WHtR – Waist-to-Height Ratio, TTE – total time to exhaustion, VO_2peak_ – peak oxygen consumption, RER_peak_ – peak respiratory exchange ratio, HR_peak_ – peak heart rate, VT_2_ – second ventilatory threshold.

Values are shown as median (lower and upper quartile).

**Table S5. 8-week body composition and graded exercise test outcomes.**

|  | **HIIT** | **VLCHF** | **VLCHF+HIIT** | **Control** |
| --- | --- | --- | --- | --- |
| VAT Mass (g) | 610 (440; 810) | 550 (410; 790) | 510 (300; 680) | 580 (440; 690) |
| VAT Area (cm^2^) | 127 (92; 168) | 114 (85; 164) | 106 (63; 142) | 120 (91; 144) |
| VAT Volume (cm^3^) | 660 (480; 880) | 600 (440; 850) | 550 (330; 740) | 630 (480; 750) |
| Body mass (kg) | 77 (74; 93) | 87 (73; 97) | 83 (65; 99) | 86 (76; 97) |
| Total Body Fat (%) | 39,7 (35,4; 42,3) | 39,4 (32,3; 43,5) | 38,8 (34,4; 43,6) | 36,6 (32,6; 42,3) |
| Trunk Fat Mass (kg) | 15,8 (11,9; 17,8) | 15,4 (12,1; 18,7) | 15,7 (11,1; 19,8) | 16,6 (13,1; 18,9) |
| Relative Trunk Fat Mass (%) | 37,6 (32,9; 41) | 37,1 (31,4; 42,4) | 37,8 (31,8; 42,4) | 37,2 (33,9; 40,3) |
| Total Lean Mass (kg) | 46 (43; 56) | 49 (43; 61) | 48 (39; 59) | 51 (44; 57) |
| Waist Circumference (cm) | 97 (91; 105) | 99 (90; 106) | 95 (85; 110) | 104 (92; 108) |
| Hip Circumference (cm) | 103,4 (101; 109,1) | 104,4 (101; 111,2) | 102,7 (98,3; 112,1) | 105,2 (100,5; 111) |
| WHR (-) | 0,9 (0,9; 1) | 0,9 (0,9; 1) | 0,9 (0,8; 1) | 1 (0,9; 1) |
| WHtR (-) | 0,6 (0,5; 0,6) | 0,6 (0,5; 0,6) | 0,6 (0,5; 0,6) | 0,6 (0,6; 0,6) |
|  |  |  |  |  |
| TTE (min:s) | 13:30 (11:28; 15:59) | 12:30 (8:46; 15:50) | 12:35 (10:45; 15:15) | 13:05 (11:09; 16:11) |
| VO_2peak_ (l/min) | 2.61 (2.41; 2.97) | 2.40 (2.11; 3.12) | 2.54 (2.10; 3.18) | 2.69 (2.14; 3.36) |
| VO_2peak_ (ml/kg/min) | 32.65 (27.85; 36.33) | 29.6 (22.75; 34.85) | 30.90 (26.25; 37.65) | 30.70 (28.40; 34.40) |
| RER_peak_ | 1.05 (1.01; 1.07) | 0.95 (0.92; 1.01) | 0.92 (0.86; 0.97) | 1.05 (1.01; 1.08) |
| HR_peak_ (bpm) | 175.0 (167.0; 187.3) | 181.5 (166.3; 194.0) | 180.5 (171.5; 187.5) | 182.5 (172.5; 189.5) |
| VT_2_ (% VO_2peak_) | 90.5 (85.8; 93.3) | 90.0 (88.0; 94.0) | 89.0 (85.3; 93.8) | 91.0 (86.0; 94.0) |

Legend*:* VAT – visceral adipose tissue, WHR – Waist-to-Hip Ratio, WHtR – Waist-to-Height Ratio, TTE – total time to exhaustion, VO_2peak_ – peak oxygen consumption, RER_peak_ – peak respiratory exchange ratio, HR_peak_ – peak heart rate, VT_2_ – second ventilatory threshold.

Values are shown as median (lower and upper quartile).

**Table S6. 12-week body composition and graded exercise test outcomes.**

|  | **HIIT** | **VLCHF** | **VLCHF+HIIT** | **Control** |
| --- | --- | --- | --- | --- |
| VAT Mass (g) | 630 (470; 760) | 490 (350; 700) | 500 (280; 640) | 600 (430; 720) |
| VAT Volume (cm^3^) | 680 (500; 820) | 530 (380; 750) | 540 (300; 690) | 650 (470; 780) |
| VAT Area (cm^2^) | 131 (97; 157) | 102 (73; 145) | 103 (58; 132) | 125 (89; 150) |
| Body mass (kg) | 77 (74; 94) | 86 (73; 95) | 82 (65; 96) | 87 (77; 96) |
| Total Body Fat (%) | 39.8 (34.8; 42.1) | 38.8 (31.9; 42.6) | 36.9 (32.8; 42.2) | 37.2 (32.9; 42.3) |
| Trunk Fat Mass (kg) | 15.4 (12.4; 17.7) | 16 (12; 18.6) | 14.1 (9.5; 18.8) | 17 (13.8; 18.9) |
| Relative Trunk Fat Mass (%) | 37.4 (33.3; 41.7) | 36.1 (30.7; 42.1) | 35.2 (29.5; 41) | 37.1 (32.2; 40.7) |
| Total Lean Mass (kg) | 46 (42; 55) | 47 (43; 61) | 47 (40; 59) | 51 (44; 61) |
| Waist Circumference (cm) | 96.0 (92.0; 104.0) | 100.0 (90.0; 104.0) | 92.0 (83.0; 106.0) | 103.0 (91.0; 111.0) |
| Hip Circumference (cm) | 103.0 (101.2; 108.5) | 104.5 (100.8; 109.7) | 102.2 (97.0; 110.6) | 105.8 (100.2; 111.4) |
| WHR (-) | 0.94 (0.90; 0.97) | 0.93 (0.89; 0.99) | 0.91 (0.85; 0.97) | 0.97 (0.92; 1.02) |
| WHtR (-) | 0.58 (0.55; 0.61) | 0.58 (0.53; 0.61) | 0.57 (0.50; 0.62) | 0.61 (0.55; 0.64) |
|  |  |  |  |  |
| TTE (min:s) | 13:47 (11:54; 16:21) | 11:37 (9:17; 15:33) | 13:39 (10:47; 15:27) | 12:59 (10:30; 15:45) |
| VO_2peak_ (l/min) | 2.55 (2.19; 2.96) | 2.44 (2.15; 3.19) | 2.60 (2.12; 2.99) | 2.49 (2.04; 3.26) |
| VO_2peak_ (ml/kg/min) | 32.75 (26.43; 34.73) | 30.80 (24.95; 35.55) | 33.25 (27.35; 36.73) | 29.90 (24.90; 33.60) |
| RER_peak_ | 1.05 (1.00; 1.09) | 0.97 (0.96; 1.01) | 0.97 (0.92; 1.01) | 1.04 (1.00; 1.09) |
| HR_peak_ (bpm) | 180.0 (169.0; 187.0) | 182.0 (162.0; 190.0) | 178.5 (169.8; 186.8) | 181.0 (168.0; 187.0) |
| VT_2_ (% VO_2peak_) | 88.0 (83.0; 94.0) | 90.0 (87.5; 93.0) | 90.0 (86.5; 93.0) | 89.0 (85.0; 92.0) |

Legend*:* VAT – visceral adipose tissue, WHR – Waist-to-Hip Ratio, WHtR – Waist-to-Height Ratio, TTE – total time to exhaustion, VO_2peak_ – peak oxygen consumption, RER_peak_ – peak respiratory exchange ratio, HR_peak_ – peak heart rate, VT_2_ – second ventilatory threshold.

Values are shown as median (lower and upper quartile).

**Table S7. Body composition and cardiorespiratory fitness differences after 4 weeks**

|  | **HIIT** | **VLCHF** | **VLCHF+HIIT** | **Control** | **Between-group diff.**  **(p-value)** |
| --- | --- | --- | --- | --- | --- |
|  | **∆*M* (95% CI)** | **∆*M* (95% CI)** | **∆*M* (95% CI)** | **∆*M* (95% CI)** |  |
| VAT Mass (g) | -24 (-54.5; 12) | -52 (-77; -33)** | -62 (-85; -22)* | 11 (-15; 35) | 0,003^b^ |
| VAT Area (cm^2^) | -4.9 (-12; 2.8) | -10.6 (-16.6; -7)** | -13 (-18; -4.6)* | 2 (-2.9; 7.1) | 0,003^b^ |
| VAT Volume (cm^3^) | -26 (-59; 13) | -56 (-83; -35)** | -67 (-91.5; -23)* | 12 (-16; 37.5) | 0,003^b^ |
| Body Mass (kg) | -0.1 (-0.9; 0.4) | -3.9 (-4.6; -3.4)** | -4 (-4.6; -3.4)** | -1 (-1.3; -0.2)* | < 0.001^a^ |
| Total Body Fat (%) | -0.4 (-0.8; 0) | 0.4 (-0.5; 0.5) | -0.3 (-0.8; 0.1) | -0.3 (-0.6; 0.2) | 0,66 |
| Trunk Fat Mass (kg) | -0.4 (-0.7; 0.1) | -1 (-1.4; -0.7)** | -1.1 (-1.5; -0.7)** | -0.3 (-0.5; 0.5) | < 0.001^a^ |
| Relative Trunk Fat Mass (%) | -0.8 (-1.1; 0.2) | -0.2 (-0.8; 0.4) | -0.5 (-1; 0.1) | -0.5 (-0.8; 0.4) | 0,788 |
| Total Lean Mass (kg) | -0.1 (-0.4; 0.4) | -2.4 (-3; -2.1)** | -1.9 (-2.7; -1.6)** | -0.1 (-0.7; 0.4) | < 0.001^a^ |
| Waist Circumference (cm) | -1.4 (-2.2; 0.2) | -4.3 (-5.1; -3)** | -4.8 (-5.9; -4.3)** | -1.1 (-2.1; 0.2) | < 0.001^a^ |
| Hip Circumference (cm) | -0.1 (-0.5; 0.3) | -1.6 (-2.2; -1.4)** | -1.8 (-2.1; -1.4)** | -0.3 (-0.7; -0.1)* | < 0.001^a^ |
| WHR (-) | -0.012 (-0.021; 0.004) | -0.023 (-0.033; -0.012)** | -0.032 (-0.038; -0.021)** | -0.003 (-0.016; 0.006) | 0,001^c^ |
| WHtR (-) | -0.008 (-0.013; 0.001) | -0.025 (-0.03; -0.017)** | -0.031 (-0.034; -0.025)** | -0.006 (-0.012; 0.001) | < 0.001^a^ |
|  |  |  |  |  |  |
| TTE (min:s) | 0:50 (-0:03; 1:15) | 0:25 (-0:25; 0:44) | 0:33 (0:08; 0:57)* | 0:02 (-0:15; 0:55) | 0.548 |
| VO_2peak_ (l/min) | 0.07 (-0.06; 0.14) | -0.15 (-0.20; 0.01) | 0.04 (-0.07; 0.19) | -0.04 (-0.13; 0.07) | 0.100 |
| VO_2peak_ (ml/kg/min) | 0.60 (-0.65; 1.80) | -0.50 (-1.35; 1.00) | 2.20 (0.05; 2.85)* | -0.20 (-1.80; 0.95) | 0.141 |
| RER_peak_ | -0.03 (-0.06; 0.02) | -0.07 (-0.10; -0.06)** | -0.10 (-0.12; -0.05)** | -0.02 (-0.05; 0.03) | 0.002^d^ |
| HR_max_ (bpm) | -2.0 (-5.5; 1.5) | 4.0 (0.5; 5.5)* | 5.0 (1.5; 7.5)* | -2.0 (-4.5; 1.5) | 0.004^a^ |
| VT_2_ (% VO_2peak_) | 1.0 (-0.5; 5.5) | -1.0 (-3.0; 2.0) | 0.0 (-3.0; 2.0) | 0.0 (-3.0; 3.5) | 0.407 |

Legend*:* VAT – visceral adipose tissue, WHR – Waist-to-Hip Ratio, WHtR – Waist-to-Height Ratio, TTE – total time to exhaustion, VO_2peak_ – peak oxygen consumption, RER_peak_ – peak respiratory exchange ratio, HR_max_ – maximal heart rate, VT_2_ – second ventilatory threshold.

Data are the median differences (∆*M*) between baseline minus 12-week measures with 95% confidence intervals (CI). The complete dataset is reported in the Supplementary Material.

Two-tailed Wilcoxon signed-rank test: * significant differences (p<0.05) for baseline vs. 12-week; ** significant differences (p<0.001) for baseline vs. 12-week.

Kruskal-Wallis test for the between-group differences. Post-hoc analysis: ^a^ – VLCHF and VLCHF+HIIT vs. HIIT and Control, ^b^ – HIIT and Control vs. HIIT and VLCHF and VLCHF+HIIT, ^c^ – Control and HIIT vs. HIIT and VLCHF vs. VLCHF and VLCHF+HIIT, ^d^ – HIIT and Control vs. VLCHF and VLCHF+HIIT vs. VLCHF+HIIT vs. Control.

**Table S8. Body composition and cardiorespiratory fitness differences after 8 weeks**

|  | **HIIT** | **VLCHF** | **VLCHF+HIIT** | **Control** | **Between-group diff.**  **(p-value)** |
| --- | --- | --- | --- | --- | --- |
|  | **∆*M* (95% CI)** | **∆*M* (95% CI)** | **∆*M* (95% CI)** | **∆*M* (95% CI)** |  |
| VAT Mass (g) | -34.5 (-49.5; 6.5) | -107 (-134.5; -66)** | -89 (-107.5; -48)** | 1 (-30; 32) | < 0.001^a^ |
| VAT Area (cm^2^) | -7 (-10; 1.3) | -21.9 (-28; -13.6)** | -19 (-22.2; -10)** | 0.3 (-6.4; 6.6) | < 0.001^a^ |
| VAT Volume (cm^3^) | -37 (-53.5; 7.5) | -115 (-146; -71.5)** | -97 (-116.5; -52.5)** | 1 (-32.5; 35) | < 0.001^a^ |
| Body Mass (kg) | 0 (-0.8; 0.5) | -4.7 (-6.5; -4.2)** | -6.8 (-7.6; -5.4)** | -0.7 (-1.6; 0.2) | < 0.001^a^ |
| Total Body Fat (%) | -0.5 (-1.3; 0.2) | -1.5 (-2.2; -1.1)** | -1.5 (-2; -1)** | -0.2 (-1; 0) | 0,006^a^ |
| Trunk Fat Mass (kg) | -0.3 (-1.1; 0) | -1.9 (-2.8; -1.6)** | -2.3 (-2.9; -1.7)** | -0.3 (-0.7; 0.2) | < 0.001^a^ |
| Relative Trunk Fat Mass (%) | -1.1 (-1.8; 0) | -2.2 (-2.9; -1.4)** | -1.5 (-2.2; -1.1)** | -0.4 (-1.2; 0.3) | 0,007^b^ |
| Total Lean Mass (kg) | -0.2 (-0.4; 0.7) | -1.9 (-2.6; -1.3)** | -2.3 (-3.3; -1.8)** | -0.5 (-1; 0.6) | < 0.001^a^ |
| Waist Circumference (cm) | -1.7 (-2.7; -0.1)* | -5.8 (-7.7; -4.4)** | -7.9 (-9; -6.6)** | -1.6 (-2.2; 0.2) | < 0.001^a^ |
| Hip Circumference (cm) | 0.2 (-0.3; 0.5) | -2.4 (-3.3; -2)** | -3.2 (-3.8; -2.5)** | -0.5 (-1.1; 0.1) | < 0.001^a^ |
| WHR (-) | -0.016 (-0.025; -0.002)* | -0.039 (-0.047; -0.019)** | -0.05 (-0.055; -0.035)** | -0.007 (-0.014; 0.009) | < 0.001^a^ |
| WHtR (-) | -0.01 (-0.016; 0)* | -0.035 (-0.045; -0.026)** | -0.045 (-0.053; -0.038)** | -0.01 (-0.012; 0.002) | < 0.001^a^ |
|  |  |  |  |  |  |
| TTE (min:s) | 1:24 (0:32; 1:45)** | 0:36 (0:01; 1:08)* | 0:55 (0:19; 1:35)** | 0:43 (-0:05; 1:05) | 0,228 |
| VO_2peak_ (l/min) | 0.13 (0.08; 0.26)** | -0.16 (-0.22; -0.01)* | -0.05 (-0.16; 0.10) | -0.06 (-0.14; 0.09) | 0,001^c^ |
| VO_2peak_ (ml/kg/min) | 2.10 (1.15; 3.25)** | 0.10 (-0.90; 1.70) | 1.50 (0.30; 3.60)* | 0.25 (-1.60; 1.40) | 0,026^d^ |
| RER_peak_ | -0.01 (-0.03; 0.04) | -0.05 (-0.10; -0.04)** | -0.09 (-0.13; -0.07)** | 0.02 (-0.01; 0.07) | < 0.001^a^ |
| HR_max_ (bpm) | -2.0 (-6.0; 0.5) | 3.5 (-2.0; 5.5) | 1.0 (-2.5; 4.0) | -1.5 (-3.5; 0.5) | 0,166 |
| VT_2_ (% VO_2peak_) | 3.0 (-0.5; 5.0) | 2.0 (-0.5; 3.5) | -1.0 (-5.5; 1.0) | 1.0 (-1.5; 6.5) | 0,111 |

Legend*:* VAT – visceral adipose tissue, WHR – Waist-to-Hip Ratio, WHtR – Waist-to-Height Ratio, TTE – total time to exhaustion, VO_2peak_ – peak oxygen consumption, RER_peak_ – peak respiratory exchange ratio, HR_max_ – maximal heart rate, VT_2_ – second ventilatory threshold.

Data are the median differences (∆*M*) between baseline minus 12-week measures with 95% confidence intervals (CI). The complete dataset is reported in the Supplementary Material.

Two-tailed Wilcoxon signed-rank test: * significant differences (p<0.05) for baseline vs. 12-week; ** significant differences (p<0.001) for baseline vs. 12-week.

Kruskal-Wallis test for the between-group differences. Post-hoc analysis: ^a^ – VLCHF and VLCHF+HIIT vs. HIIT and Control, ^b^ – HIIT and Control vs. VLCHF vs. VLCHF and VLCHF+HIIT vs. HIIT and VLCHF+HIIT, ^c^ – HIIT vs. VLCHF and VLCHF+HIIT and Control, ^d^ – HIIT vs VLCHF and Control vs. VLCHF and VLCHF+HIIT and Control vs. HIIT and VLCHF+HIIT.
